# Implementing Somalia's National Transformation Plan 2025–2029 for health-system reform: a policy and practice review of governance, financing, and institutional readiness

**DOI:** 10.3389/frhs.2026.1934347

**Published:** 2026-09-30

**Authors:** Nor Haji Osman, Dahir Osman Mohamed, Hamdi Mohamud Osman, Abdifatah Nour Rage, Abdiweli Mohamed Abdi, Mohamed Ismael Iman, Aweis Ahmed Moallim, Abdirahman Mohamed Jimale, Mohamed Ahmed Ali, Ismail Dahir Ahmed, Abdikarim Abdi Adam, Abdirahman Ahmed Mohamud, Siham Mohamed Dhakane, Osman Mohamed Mohamud

**Affiliations:** 1 Department of Public Health, Faculty of Health Sciences, Al Hayat Medical University, Mogadishu, Somalia; 2 Department of Policy and Planning, Directorate of Health and Human Services, Banadir Region, Mogadishu, Somalia; 3 Noncommunicable Diseases Section, Ministry of Health and Human Services, Mogadishu, Somalia; 4 Department of Social Sciences, Faculty of Humanities and Social Sciences, Salaam University, Banadir Region, Mogadishu, Somalia; 5 Department of Medicine, Faculty of Medicine and Surgery, Al Hayat Medical University, Mogadishu, Somalia; 6 Department of Administration, Human Resources and Capacity Building, Directorate of Health and Human Services, Banadir Region, Mogadishu, Somalia; 7 Department of Environmental Health and Climate, IPC Section, Federal Ministry of Health and Human Services, Mogadishu, Somalia; 8 Department of Health, Directorate of Health and Human Services, Banadir Region, Mogadishu, Somalia; 9 Department of Public Health, Faculty of Health Sciences, Zamzam University of Science and Technology, Banadir Region, Mogadishu, Somalia; 10 Faculty of Medicine and Health Sciences, Zamzam University of Science and Technology, Mogadishu, Somalia

**Keywords:** governance, health financing, health-system reform, implementation readiness, national transformation plan, Somalia

## Abstract

Somalia's National Transformation Plan (NTP) 2025–2029 presents an ambitious health-reform agenda centred on maternal and child health, workforce development, health-system strengthening, regulation, financing, digitalisation, and emergency preparedness. The policy challenge is whether the institutional conditions required for implementation can be assembled in a fragile, federal, highly aid-dependent and predominantly mixed-provider health system. This Policy and Practice Review critically examines the implementation readiness of the NTP health chapter and proposes a sequenced framework for converting national commitments into measurable results. A structured narrative review was undertaken from June to July 2026 using peer-reviewed literature, national policy documents, and reports from governmental and international institutions. Evidence was interpreted through the health-policy triangle, the updated Consolidated Framework for Implementation Research (CFIR), applied prospectively to anticipated determinants, and a health-system resilience lens. The synthesis generated six interdependent proposed readiness foundations: a negotiated federal-state governance compact; a technically validated, costed and sequenced operational plan; sustainable financing and strategic purchasing; workforce and regulatory capacity; integrated information, monitoring and public accountability systems; and shock-responsive adaptive management. Important policy strengths include explicit targets for service coverage, workforce expansion, hospital rehabilitation, regulation and digital health. Key risks include inconsistent or under-specified targets and baselines, uncertain fiscal pathways, weak federal-state role specification, and dependence on external resources and non-state providers. Comparative evidence suggests that contracting and pooled financing can expand services when stewardship, fiduciary safeguards, workforce integration, community accountability and learning systems develop concurrently. We propose a phased 2026–2029 pathway prioritising technical validation, essential-service continuity, subnational compacts, workforce payroll transition, interoperable data systems and annual public performance reviews. The proposed framework is prospective and has not yet been empirically validated; consultation with federal and subnational authorities, providers, partners, civil society and communities is required before formal adoption.

## 1 Introduction

Somalia entered the 2025–2029 planning period with a health system shaped by prolonged conflict, recurrent climate shocks, large-scale displacement, a constrained public revenue base, fragmented external assistance, and a substantial private and non-governmental service-delivery sector. The National Transformation Plan (NTP) reframes health as a central component of social and human-capital transformation and sets quantified ambitions for service coverage, mortality reduction, workforce expansion, infrastructure, regulation, health financing, digitalisation and emergency preparedness (1). It builds on a layered policy architecture: the Ninth National Development Plan supplied the preceding national development framework and implementation lessons; HSSP III set the 2022–2026 sector strategy; the revised EPHS defined the priority service package and resource requirements; the health-sector investment case translated that package into investment priorities; and the UHC roadmap set the direction for service coverage and financial protection (2–5). The NTP consolidates these strands within a higher-level transformation agenda, but does not replace the need for a costed operational health-sector plan.

The health chapter of the NTP is unusually explicit for a national development plan. It aims to increase access to essential health services, raise DTP3 immunisation coverage, rehabilitate regional hospitals, establish referral hospitals, expand the health workforce, operationalise professional and medicines regulation, increase government health expenditure to 10% of the national budget, expand health insurance and strengthen monitoring, digital health and public-health emergency systems (1). These commitments provide a useful political mandate. They also create a demanding implementation agenda that extends beyond the present administrative, fiscal and regulatory capacity of the sector.

The implementation challenge is compounded by severe health need. National and international sources consistently report low coverage of essential services, substantial geographic and financial barriers, high maternal and child mortality, major malnutrition and immunisation gaps, and a critically constrained and unevenly distributed workforce (1, 6–9). Recent Somalia-specific research has also documented fragmented federal-state responsibilities, the influence of external actors, limited regulatory reach, weak financial protection and continuing dependence on parallel delivery systems (10–18). These conditions mean that implementation cannot be understood as a linear process of moving activities from a policy document into annual workplans. It is a political, institutional and adaptive process involving multiple centres of authority and financing.

This review is framed as a Policy and Practice Review focused on implementation readiness. Rather than asking whether the NTP's objectives are desirable, it asks whether the arrangements needed to deliver them are sufficiently coherent, financed, governed, monitored and resilient. This distinction is important because fragile-state reforms often fail not from lack of technical recommendations, but from unclear authority, misaligned incentives, fragmented financing, insufficient implementation capacity and weak mechanisms for learning and accountability (19–24).

The review has three objectives: first, to examine the coherence of the NTP health commitments with Somalia's existing health-sector policy architecture; second, to synthesise evidence on the governance, financing, workforce, regulatory, information and resilience factors that will shape implementation; and third, to propose a sequenced, actor-specific implementation-readiness framework for 2026–2029. The analysis is intended to support Somali health authorities, federal member states (FMS), Banadir Regional Administration, development partners, service providers, civil society and researchers engaged in translating the NTP into operational programmes.

Selected baselines illustrate the scale of the challenge. Table 1 compares selected baselines with the NTP's 2029 targets and shows the change required. The latest sources used in this review report a UHC service-coverage index of 33.5 (2021 estimate), skilled birth attendance of 32%, maternal mortality of 692 per 100,000 live births, neonatal mortality of 38 per 1,000, infant mortality of 61 per 1,000 and under-five mortality of 122 per 1,000 (2020 estimates) (1, 6, 14). The 2024 WHO/UNICEF estimate placed DTP3 coverage at 42%. The NTP reports that acute malnutrition affects more than 1.5 million children and that workforce density is 7.91 health workers per 10,000 population, although it does not specify reference years for these two planning baselines (1, 25). Financial protection is weak: out-of-pocket payments account for 44.9% of total health expenditure, while the 2021 resource-mapping report estimated that donors and households each financed approximately 45% of health spending (17, 26). These estimates are not fully contemporaneous and should therefore be treated as the best available reference points rather than a single harmonised baseline year.

**Table 1:** Selected baselines and change required to reach the principal NTP mortality and service-coverage targets.

Indicator	Baseline and year	2029 NTP target	Absolute change required	Relative change required
UHC service-coverage index	33.5 (2021) (14)	40	+6.5 points	+19.4%
Maternal mortality ratio	692 per 100,000 (2020) (1, 6)	<400	Reduction of at least 292	At least 42.2% reduction
Neonatal mortality rate	38 per 1,000 (2020) (6)	<20	Reduction of at least 18	At least 47.4% reduction
Infant mortality rate	61 per 1,000 (2020) (1, 6)	<37	Reduction of at least 24	At least 39.3% reduction
Under-five mortality rate	122 per 1,000 (2020) (1, 6)	<60	Reduction of at least 62	At least 50.8% reduction

## 2 Review approach

### 2.1 Design and analytical orientation

This article used a structured critical narrative review and policy-analysis design. A narrative approach was selected because the evidence base combines national plans, implementation reports, financing documents, qualitative studies, surveys, reviews and comparative fragile-state evidence that cannot be meaningfully reduced to a single intervention-effect estimate. The review was informed by the Scale for the Assessment of Narrative Review Articles (SANRA), particularly the requirements for a clear aim, transparent search description, appropriate referencing, scientific reasoning and presentation of relevant evidence (27).

The analytical framework combined three perspectives. First, the Walt-Gilson health-policy triangle was used to assess policy content, context, actors and process (28). Second, the updated Consolidated Framework for Implementation Research (CFIR 2.0) was applied prospectively to assess anticipated implementation determinants rather than retrospectively to evaluate an implemented intervention (29). In CFIR terminology, the innovation domain was defined as the operational package needed to implement the NTP health chapter—not the entire national plan as a single intervention—while the outer setting, inner setting, individuals and implementation process were examined as interacting determinants. Third, health-system resilience was treated as the capacity to absorb shocks, adapt operations and transform institutions while preserving equitable essential services (20, 21, 30, 31). These perspectives were integrated into an implementation-readiness matrix rather than applied as separate checklists.

### 2.2 Search strategy and information sources

Searches were conducted from June to July 2026 using Google Scholar as the multidisciplinary scholarly index, PubMed and PubMed Central, targeted publisher searches, and institutional repositories. Search concepts combined Somalia with health system, health policy, NTP, HSSP, EPHS, governance, federalism, financing, aid, out-of-pocket expenditure, workforce, private providers, information systems, accountability, resilience and fragile or conflict-affected settings. Comparative searches covered Afghanistan, Ethiopia, Liberia, Rwanda and Sierra Leone where reform experience addressed contracting, decentralisation, community health, pooled financing, workforce integration or post-conflict recovery. South Sudan was removed from the stated comparative scope because no South Sudan source was retained in the final synthesis. Because the search was purposive and iterative, database-level yield counts were not retained.

Grey literature was purposively retrieved from the Federal Government of Somalia, the Federal Ministry of Health, the Ministry of Planning, the Somali National Bureau of Statistics, WHO, UNICEF, the World Bank, the Global Financing Facility and related institutions. Core documents included the NTP 2025–2029, HSSP III, the NDP-9 mid-term review, the EPHS investment case, the UHC roadmap, health-sector resource mapping and expenditure tracking, partnership alignment principles, national survey data and global monitoring reports (1–8, 25, 26, 32–34).

### 2.3 Eligibility, evidence weighting and synthesis

Eligible sources were English-language documents published primarily from 2015 to 2026 that addressed Somalia's health-system policies, implementation conditions or relevant experience in fragile and conflict-affected settings. Earlier foundational sources were retained where conceptually necessary. Titles, abstracts or executive summaries were screened for relevance, followed by full-text assessment of potentially eligible records. Sources were excluded when provenance could not be verified, when they duplicated a more authoritative source, or when they did not directly inform implementation. One author conducted the initial screening and evidence charting; potentially eligible sources, domain assignments and interpretive differences were checked through author-team review and resolved by consensus. The final purposive corpus comprised 60 cited sources. Because this was a structured narrative rather than systematic review, a PRISMA flow diagram and exhaustive database-yield accounting were not used; this limitation is stated explicitly.

Throughout the synthesis, Somalia-specific evidence is cited as the basis for descriptive or causal interpretations; comparative evidence is labelled by setting and accompanied by transferability limits; and normative recommendations are signalled with terms such as ‘we propose’, ’should’ or ‘would’. The readiness foundations and phased pathway are therefore analytical proposals generated by triangulating these three evidence streams, not empirically validated requirements.

Evidence was charted under eight domains: policy coherence; federal and institutional governance; financing and purchasing; service delivery and infrastructure; workforce and regulation; private-sector and partner engagement; data, monitoring and accountability; and emergency preparedness and resilience. Greater weight was given to official national documents for policy commitments, nationally representative data for population indicators, peer-reviewed empirical studies for implementation mechanisms, and systematic or scoping reviews for cross-setting conclusions. Where estimates differed, the review reports the source and year rather than presenting an artificial single figure. Because this was a policy and practice review rather than a systematic review of intervention effectiveness, no meta-analysis or formal pooled risk-of-bias score was undertaken.

## 3 The NTP health agenda and its policy architecture

The NTP organises the health agenda around three key result areas: strengthening maternal and child health services, developing a qualified medical workforce and strengthening health systems (1). Its enabling agenda includes sustainable financing, professional and pharmaceutical regulation, monitoring and evaluation, digital health information systems, and emergency preparedness and research. The plan targets UHC service coverage of 40% by 2029, maternal mortality below 400 per 100,000 live births, neonatal mortality below 20 per 1,000, infant mortality below 37 per 1,000 and under-five mortality below 60 per 1,000 (1). Chapter 15 (pp. 425–435) specifies the following headline targets: 80% DTP3 coverage; rehabilitation of 18 regional hospitals; establishment of state referral hospitals; workforce expansion; and 35% health-insurance penetration, alongside private-investment and regulatory milestones (1). Page-level pointers are provided because these targets appear in figures and initiative tables as well as narrative text.

This broad architecture is aligned with HSSP III and the EPHS in its emphasis on primary health care, reproductive, maternal, newborn, child and adolescent health, nutrition, communicable disease control, essential medicines, workforce, information and governance (2, 4, 10). It also aligns with the UHC roadmap's focus on expanding service coverage and financial protection (5). The NTP's added value is political visibility, multisectoral positioning and a clearer orientation toward system transformation. Its weakness is that a national transformation plan does not itself provide the operational detail required for implementation. It must be translated into a technically validated health-sector implementation plan that reconciles existing policies, updates baselines, identifies costs and financing sources, clarifies roles, and defines annual milestones. Table 2 summarises the alignment of the NTP health agenda with Somalia’s existing policy instruments. The emphasis on integrated system functions and primary-health-care implementation is also consistent with established WHO operational frameworks (35, 36).

**Table 2:** Alignment of the NTP health agenda with Somalia's existing policy architecture.

Policy instrument	Primary contribution	Alignment with NTP	Implementation issue requiring resolution	Practical implication
NTP 2025-2029 (1)	Political mandate, quantified transformation targets and cross-sector framing.	Defines three key result areas and five major enablers for health.	Contains some inconsistent or insufficiently specified targets, baselines, sequencing and financing assumptions.	Undertake a formal technical validation and issue a consolidated health implementation annex.
HSSP III 2022-2026 (2)	Sector strategy for resilient, equitable and quality services.	Provides the sectoral foundation for governance, service delivery, workforce, medicines and information systems.	Ends before the NTP; indicators and institutional arrangements require transition to a successor strategy.	Use 2026 as a bridge year and develop HSSP IV aligned to the NTP through 2029/2030.
NDP-9 and mid-term review (3)	Previous national planning and implementation lessons.	Identifies recurring weaknesses in coordination, indicators, accountability and delivery.	Lessons risk being repeated if the NTP relies on broad targets without implementation ownership.	Translate lessons into role matrices, quarterly milestones and public review mechanisms.
EPHS and Investment Case (4, 10)	Defines priority services and indicative resource needs.	Provides the service-delivery content for the NTP's UHC and primary-care commitments.	Coverage expansion is constrained by funding, workforce, commodities and inconsistent provider arrangements.	Make EPHS delivery the unit of planning, contracting, costing and performance measurement.
UHC Roadmap (5)	Sets direction for coverage and financial protection.	Supports the NTP coverage and insurance ambitions.	Insurance expansion may be premature without revenue, pooling, purchasing, provider payment and regulatory foundations.	Prioritise tax-funded essential services and targeted protection before large-scale contributory insurance.
Partnership alignment principles (34)	Country leadership, harmonisation and mutual accountability.	Supports donor alignment, unified monitoring and reduced parallelism.	Voluntary commitments may not change financing behaviour without measurable compliance indicators.	Publish an annual partner-alignment scorecard linked to the financing map and NTP results.

## 4 Critical implementation-readiness findings

Table 3 summarises major NTP commitments, implementation assumptions and readiness tests.

**Table 3:** Major NTP commitments, implementation assumptions and readiness tests.

NTP commitment	Underlying assumption	Principal risk	Readiness test before scale-up	Suggested performance evidence
Increase UHC service coverage to 40% by 2029 (1)	Coverage can expand through combined public, private and partner delivery.	Baseline inconsistency; expansion without quality or financial protection.	Define tracer services, denominator, data source, quality threshold and equity disaggregation.	Service coverage index plus effective coverage, travel time, utilisation and catastrophic spending.
Government health spending reaches 10% of national budget (1)	Fiscal space and political prioritisation will increase.	Budget not released or executed; recurrent services crowded out by capital spending.	Agree annual nominal pathway, protected programme lines and expenditure tracking.	Approved, released and executed expenditure; per-capita public spending; primary-care share.
Rehabilitate 18 regional hospitals and establish referral hospitals (1)	Capital investment will generate functional access and quality.	Inconsistent referral-hospital count; insufficient recurrent budget, staff and maintenance.	Complete needs, location, recurrent-cost, workforce and referral-network assessments.	24/7 readiness, staffing, WASH/power uptime, essential diagnostics, referral completion and quality outcomes.
Reach 95% staffing fulfilment and expand primary-care workforce (1)	Training and recruitment can close gaps.	No funded establishment; maldistribution, attrition and donor-payroll dependence.	Verify facility-based establishment, payroll cost, deployment and retention package.	Filled funded posts, rural retention, absenteeism, competency and supervision indicators.
Achieve 35% health-insurance penetration (1)	Private or contributory insurance can rapidly improve protection.	Large informal sector, adverse selection, fragmented risk pools and exclusion of the poor.	Complete financing feasibility, benefits, subsidy, purchasing and regulation design.	Population coverage with effective benefits, claims ratios, OOP reduction and equity.
Operationalise professional and medicines regulation (1)	Regulatory bodies can rapidly license and enforce standards.	Insufficient finance and inspectors; fragmented legal recognition; regulatory capture.	Enact regulations, approve governance safeguards, digital registries and phased inspection plans.	Registered workforce/facilities/products, inspection coverage, enforcement timeliness and public disclosure.
Digitise and integrate health information systems (1)	Technology will improve decisions and accountability.	Parallel digital platforms, poor interoperability, low data quality and privacy risks.	Approve enterprise architecture, data standards, governance, infrastructure and workforce plan.	Completeness, timeliness, concordance, interoperability transactions and documented data-use decisions.

### 4.1 Policy coherence and technical validation

The first readiness requirement is a single technically coherent implementation specification. The NTP provides a strong political narrative but contains details that need reconciliation before budgets and contracts are built around them. For example, an earlier summary refers to constructing seven state referral hospitals, whereas the detailed health chapter proposes eight referral hospitals. The specialist-development section repeats a target for 4,000 primary-care workers that appears to belong to the preceding workforce initiative. Several activities are stated to begin in January 2025 even though the NTP was publicly launched in March 2025. These inconsistencies appear in the Chapter 15 initiative summaries and results framework (pp. 425–435). They do not invalidate the strategy, but they illustrate the risk of treating a high-level planning document as an operational plan (1).

Technical validation should therefore precede large financial commitments. Each NTP target should have a confirmed baseline, numerator and denominator, geographic scope, responsible institution, annual milestone, verified cost, financing source, procurement route, implementation dependency and reporting mechanism. Targets that are politically important but fiscally uncertain should be expressed as phased scenarios rather than fixed promises. A national implementation annex, jointly endorsed by the Federal Ministry of Health, FMS ministries, Banadir and the Ministry of Finance, would provide a stable reference for partners and providers.

### 4.2 Federal governance, role clarity and stewardship

Somalia's federal structure is both a constraint and an opportunity. It enables adaptation to diverse subnational conditions, but the distribution of authority for policy, standards, financing, workforce management, regulation, procurement and service delivery remains contested or inconsistently applied. Research on health-sector federalism in Somalia describes overlapping mandates, variable state capacity and the continuing influence of donor and implementing-agency structures (11–14). Similar problems were highlighted during implementation of earlier plans (3). These stewardship challenges are consistent with established health-system governance dimensions (37).

The appropriate response is not recentralisation. It is a negotiated governance compact that specifies which functions require national uniformity and which should be decentralised. The Federal Ministry of Health should initiate the process jointly with FMS health ministries and Banadir, while the federal ministries responsible for finance, planning and intergovernmental affairs should participate where fiscal transfers or legal authority are concerned. Political authorisation should come through the established intergovernmental health coordination mechanism and, where required, cabinet or legislative endorsement. Participation incentives include predictable and formula-based transfers, access to aligned partner financing, shared procurement and technical support, recognition of subnational planning authority, and transparent performance information. The compact should define mediation, escalation and periodic renegotiation procedures for disputes over authority, financing, data and accountability; independent facilitation or an agreed intergovernmental committee may be used when bilateral resolution fails. Enforcement should rely on published commitments, conditional financing and joint review rather than federal direction alone. National functions should include policy, standards, regulation, national surveillance, pooled procurement frameworks, health accounts, core information standards and interstate equity mechanisms. FMS and Banadir should lead contextualised planning, facility oversight, workforce deployment, supervision, community accountability and district-level performance improvement. Joint functions should include costing, emergency response, referral networks, partner coordination and annual sector review.

Stewardship also requires the government to govern a mixed-provider system rather than assume that public facilities alone will deliver the NTP. Private, faith-based, non-governmental and community providers already deliver substantial care, especially in urban areas and donor-supported programmes. Evidence from low-income settings suggests that private providers can extend access but may worsen financial protection and quality inequities when regulation and purchasing are weak (18, 38). Government leadership should therefore be exercised through licensing, service standards, information requirements, contracting, referral rules, quality assurance and strategic purchasing.

### 4.3 Financing, pooling and strategic purchasing

Financing is the most immediate constraint on the NTP. Somalia's health system remains heavily dependent on external assistance and household payments, while public health allocations remain low relative to need (14–17, 26). A recent household study reported that out-of-pocket payments represented 44.9% of total health expenditure and that insurance coverage was extremely limited; the same study found substantial catastrophic spending and harmful coping strategies (17). Although exact estimates vary by year and method, the direction is consistent: households bear excessive financial risk and the public financing base is insufficient.

The NTP's 35% health-insurance penetration target (Chapter 15, pp. 425–435) is not operationally defined in the source plan. The plan does not state clearly whether the denominator is the total population, households, workers or another eligible group; nor does it specify the mix of contributory and subsidised arrangements, the guaranteed benefit package, subsidy source, pooling structure, purchasing agency or provider-payment method (1). These design choices should be resolved through an actuarial, fiscal and equity assessment. Until then, the 35% figure should be treated as a policy aspiration, not a measurable financial-protection outcome. Reporting should distinguish enrolment, active entitlement, services actually covered, out-of-pocket expenditure and catastrophic spending.

The NTP's commitment to raise government health spending to 10% of the national budget is politically valuable but requires a credible fiscal pathway (1). The target is below the 15% Abuja benchmark and should not be interpreted as automatic evidence of adequacy (32). More importantly, a budget-share target alone does not guarantee execution, equity or value. The implementation plan should specify annual nominal allocations, release rates, executed expenditure, subnational distribution, programme composition and the share financing essential services, workforce, commodities, public health and system functions. Protecting a defined minimum package from in-year cuts may be more consequential than announcing a percentage target.

A rapid shift of all donor resources into government systems would carry fiduciary and continuity risks. Conversely, maintaining parallel projects will continue to fragment planning, payroll, commodities, reporting and accountability. A transitional financing model is therefore required: one national costed plan; a comprehensive financing map; common programme and geographic classifications; aligned results and audit requirements; and progressively pooled or pooled-equivalent arrangements with independent fiduciary safeguards. Funds need not immediately enter a single treasury account to be aligned, but every major financing stream should be visible, mapped to NTP outputs and reviewed jointly.

Strategic purchasing should become the practical bridge between financing and service delivery. Contracts with public, private and non-governmental providers should specify the EPHS services to be delivered, catchment population, equity obligations, workforce and quality standards, commodity responsibilities, data reporting, payment method, verification and remedies for underperformance. Afghanistan demonstrates that contracting can expand access in a fragile setting, but also that dependence on external financing and political shocks can rapidly reverse gains (23, 39, 40). Somalia should therefore avoid building a purchasing model that the state cannot eventually finance, regulate or monitor.

### 4.4 Service-delivery prioritisation and infrastructure realism

The NTP combines primary-care, maternal and child health, nutrition, immunisation, hospital and specialised-service ambitions. The risk is that visible capital investments may displace less visible but more consequential recurrent functions. Hospital rehabilitation and referral capacity are important, particularly for emergency obstetric, neonatal, trauma and diagnostic care. However, construction without staffing, power, water, medicines, maintenance, referral transport, blood, laboratory quality and recurrent budgets produces nominal rather than functional capacity.

Implementation should use the EPHS and referral pathway as the organising logic. The first priority is reliable access to a defined set of essential services at community and primary-care levels, linked to functional first-referral and regional hospitals. Facility investment should be selected using population, travel-time, disease burden, displacement, service utilisation, security, climate exposure and existing-provider capacity. Every capital project should include a ten-year recurrent-cost and maintenance assessment and should not proceed without an approved staffing and operating model.

Maternal and child health illustrates the need for system integration. Improving facility delivery requires more than maternity wards: community trust, financial access, antenatal risk detection, skilled staff, blood and medicines, communication, referral transport, surgical capacity, newborn care, postnatal follow-up and data review are all necessary. Recent analysis of the 2020 Somali Health and Demographic Survey confirms that facility delivery remains socially and geographically patterned, reinforcing the need to link coverage targets with equity-focused community, referral and financial-access interventions (41). Comparative evidence from fragile settings likewise shows that political prioritisation is strengthened when local champions, community and civil-society actors, technical plans and financing are aligned (19). Vertical campaigns can produce gains, but durable mortality reduction depends on reliable service systems. Additional Somalia-specific evidence on institutional delivery and humanitarian reproductive-health programmes supports the need for geographically responsive referral and service-delivery arrangements (42, 43).

### 4.5 Workforce, professional regulation and institutional capacity

Workforce shortage and maldistribution are binding constraints. The NTP reports 7.91 health workers per 10,000 population and proposes 95% staffing fulfilment in public facilities, large-scale primary-care recruitment, specialist training and stronger professional regulation (1). Other recent analyses report even lower densities depending on the cadre definition and data source (13–15). These variations reinforce the need for a verified national health-workforce account rather than reliance on isolated estimates.

A national workforce plan should connect training, accreditation, recruitment, payroll, deployment, retention, supervision and career progression. Expanding graduates without funded positions may increase unemployment or migration rather than service availability. The plan should identify funded posts by facility and cadre, harmonise job classifications and salary scales, prioritise female and community-based workers, create hardship and housing incentives for underserved areas, and integrate donor-funded workers into transparent payroll arrangements over time. The experiences of Ethiopia and other settings show that community and primary-care workforce expansion can improve access when roles, supervision, supplies, referral and financing are institutionalised (20, 44, 45).

The National Health Professionals Council and the National Medicines Regulatory Authority are central to NTP credibility. Their mandates should be backed by legislation, predictable financing, transparent governance, conflict-of-interest controls, digital registries, inspection capacity and public reporting. Recent Somalia-focused AMR analyses describe continuing surveillance, laboratory, stewardship and regulatory gaps, and argue for coordinated human, animal and environmental action (46–48). These findings strengthen the case for linking medicines regulation with antimicrobial stewardship, diagnostic capacity, infection prevention, surveillance and a practical One Health coordination mechanism. Regulation should be risk-based and phased: high-risk facilities, products and professions first; national minimum standards; mutual recognition across FMS; and progressive enforcement that does not abruptly remove scarce providers without replacement. Training institutions should be linked to accreditation outcomes and workforce needs rather than regulated only through one-time licensing.

### 4.6 Information systems, monitoring and public accountability

The NTP correctly identifies unified monitoring and digital health as key enablers (1). Somalia has expanded DHIS2 and disease-surveillance systems, and recent studies show growing interest in data use and digital platforms (13–15). Nevertheless, digitisation can reproduce fragmentation when programmes maintain separate identifiers, forms, dashboards, servers, data standards and reporting obligations. The objective should be an interoperable national information architecture, not the number of digital applications.

A minimum architecture should include a national facility registry, workforce registry, master indicator dictionary, unique programme and geographic codes, data-exchange standards, role-based access, data-quality protocols, privacy and security governance, and defined interoperability between DHIS2, surveillance, immunisation, laboratory, logistics, civil registration, workforce, regulatory and financial systems. Private and contracted providers should be included through proportionate reporting requirements and technical support. Data collection should be reduced where indicators are not used for decisions.

Monitoring should distinguish inputs, implementation processes, outputs, coverage, quality, equity, financial protection and outcomes. Quarterly reviews should focus on a small set of actionable indicators, disaggregated by state, district, sex, age, displacement status and service-delivery channel where feasible. Recent Somalia-specific commentary identifies structured district- and regional-level supportive supervision as a practical mechanism for strengthening facility HMIS data quality and use (49). Such supervision should combine verification, coaching, documented corrective actions and follow-up rather than operate as inspection alone. Annual national health-sector reviews should publish performance, financing, partner alignment, audit findings and corrective actions. Social accountability mechanisms, including facility committees, community scorecards, complaints systems and public dashboards, can improve responsiveness, but only when institutions have resources and authority to respond (50–52).

### 4.7 Resilience, emergency preparedness and adaptive implementation

Somalia's health reform will be implemented under recurrent shocks rather than after fragility has ended. Drought, flooding, displacement, outbreaks, insecurity, supply disruption and external funding volatility must therefore be incorporated into routine design. Recent Somalia-focused analyses describe a reinforcing drought-disease cycle and persistent waterborne-disease risks in Mogadishu, highlighting how water insecurity, displacement, fragile sanitation and disrupted services translate climate shocks into public-health emergencies (53, 54). These sources are commentaries rather than effect evaluations, but they reinforce the operational case for climate-sensitive surveillance, WASH continuity, pre-positioned supplies and cross-sector coordination. Reviews of fragile and conflict-affected settings show that health-system strengthening is most effective when governance, workforce, services, supplies, infrastructure, community trust and financing are addressed as an interacting system (20, 21). Resilience should not be treated as a separate emergency project. National facility studies also document substantial gaps in infection-prevention monitoring and healthcare-waste management that may amplify service disruption and transmission risks during shocks (55–57).

The NTP's proposals to expand public-health emergency operations centres, laboratories, surveillance, risk communication and research are important (1). Implementation should link these assets to routine systems: the same workforce, laboratories, logistics, information standards and financing mechanisms should support both daily services and surge response. Facilities and supply chains should be assessed for climate and security risk. Pre-arranged emergency procurement, contingency financing, decentralised stocks, referral continuity and staff protection plans should be incorporated into contracts and facility standards.

Adaptive implementation means that annual targets and resource allocations can change when evidence, shocks or feasibility change. This is not policy failure; it is responsible management. The NTP should use explicit decision rules for reprioritisation, document why changes are made, and protect core services and vulnerable groups. A learning system requires routine data, implementation research, rapid evaluation, feedback from frontline staff and communities, and a forum with authority to make corrections (31, 58, 59).

## 5 Lessons from other fragile and reforming health systems

Comparative experience is useful only when mechanisms and contextual differences are made explicit. Somalia should not copy institutional forms from more centralised, fiscally capable or politically stable states. The transferable lesson is usually a design principle, not an entire programme. Table 4 presents comparative lessons and their limits of transferability to Somalia.

**Table 4:** Comparative lessons and limits of transferability.

Setting/reform	Relevant mechanism	Evidence-informed lesson	Transferability to Somalia	Safeguard required
Afghanistan: contracting NGOs for basic services (23, 39, 40)	Government defines package and contracts non-state providers, with performance monitoring.	Contracting can expand access where state delivery capacity is limited.	High for EPHS purchasing, particularly underserved areas.	Diversified financing, government contract-management capacity, data verification and continuity plans for political/funding shocks.
Ethiopia: health extension and community workforce (44, 45)	Large-scale community workforce with standard roles, supervision and referral.	Primary-care expansion requires institutionalised training, supplies, supervision and career pathways.	Moderate; Somalia's security, mobility and federal governance require local adaptation.	Funded posts, female-worker safety, community acceptance, referral functionality and flexible deployment.
Rwanda: coordinated planning and accountability (60)	Strong central stewardship, performance systems and partner alignment.	Clear priorities and routine review can reduce fragmentation and sustain focus.	Principles are transferable; centralised governance model is not.	Federal-state compact, independent verification and protection against coercive target management.
Liberia and Sierra Leone: post-crisis system rebuilding (20–23)	Use of recovery plans to integrate resilience, workforce and primary care.	Crisis recovery is stronger when investments build routine institutions rather than parallel emergency systems.	High for laboratory, surveillance, supply and workforce integration.	Long-term financing, local ownership and retention of surge capacities.
Cross-FCAS evidence (20–22)	Context-specific, coordinated and comprehensive health-system strengthening.	Single-component reforms are unlikely to produce durable gains in complex fragile systems.	Very high as a design principle.	Equity analysis, community involvement, conflict sensitivity and adaptive management.

## 6 Proposed implementation-readiness framework

Figure 1 presents six readiness foundations proposed from the synthesis; they have not been empirically validated. Table 5 presents the proposed phased implementation pathway for July 2026–December 2029. We first charted the evidence across eight substantive domains. The health-policy triangle was then used to connect policy content with context, actors and process; prospective CFIR constructs were used to group anticipated determinants affecting the operational package; and the resilience lens was used to test whether each function could absorb, adapt to and learn from shocks. Overlapping categories were consolidated: federal governance and actor/process findings formed Foundation 1; policy coherence, costing and sequencing formed Foundation 2; financing and purchasing formed Foundation 3; workforce, regulation, service-delivery and supply constraints formed Foundation 4; information and accountability formed Foundation 5; and emergency preparedness and cross-domain adaptive capacity formed Foundation 6. Thus, the synthesis generated a parsimonious analytical framework rather than identifying six empirically proven requirements. The foundations are interdependent and require validation through structured consultation with federal authorities, FMS, Banadir, providers, partners, civil society and communities before adoption or scale-up.

**Figure 1:**
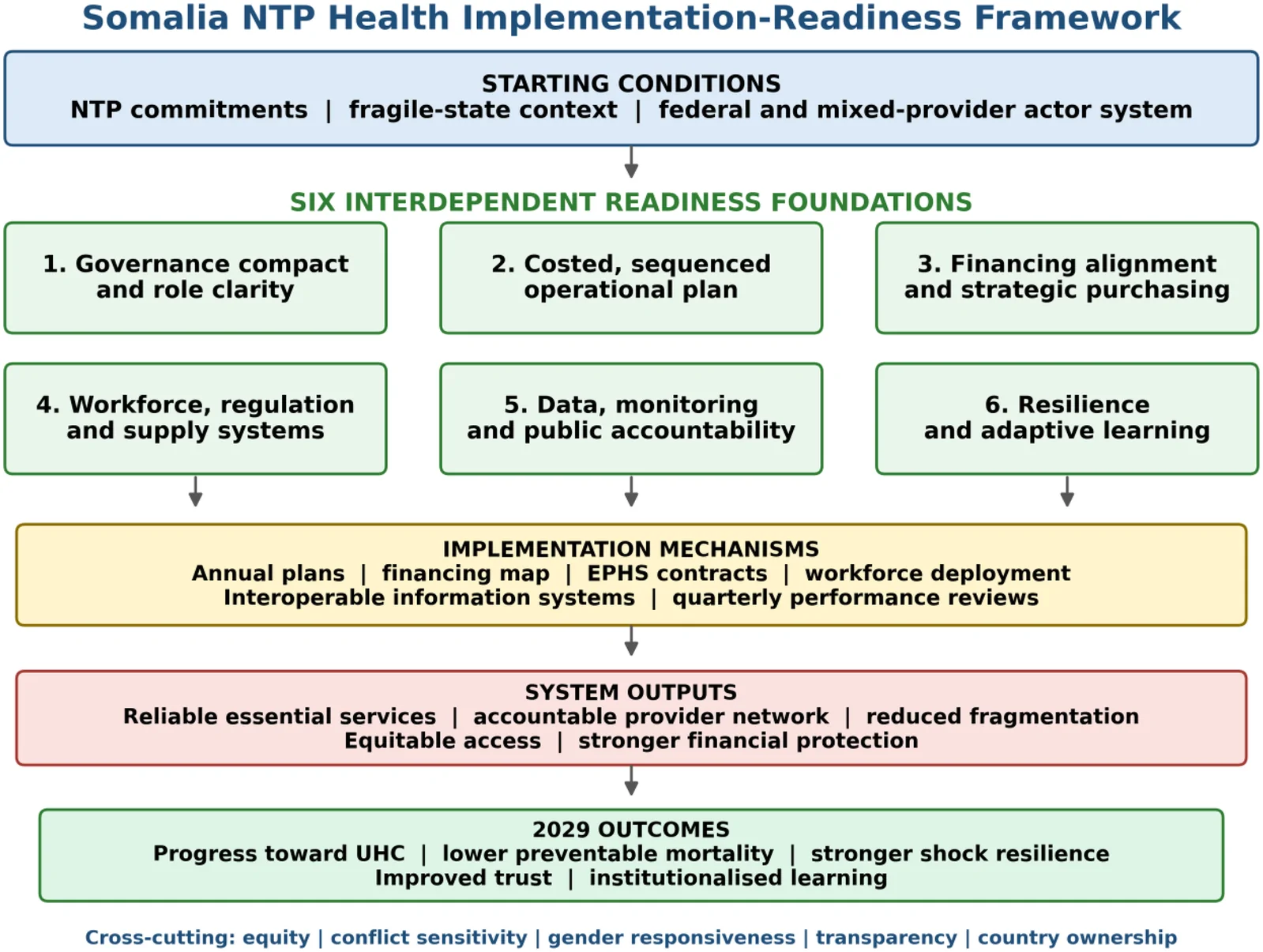
Somalia NTP health implementation-readiness framework.

**Table 5:** Proposed phased implementation pathway, July 2026-December 2029.

Phase	Priority actions	Decision products	Minimum evidence of progress	Main risk to manage
Phase 1: Technical reset and protection of essential services (Jul-Dec 2026)	Validate targets and baselines; reconcile NTP/HSSP/EPHS; map financing and actors; identify essential-service continuity risks; agree federal-state governance process; initiate HSSP IV.	Validated implementation annex; financing map; role matrix; 2027 costed annual plan; continuity and contingency plan.	All major targets have definitions, owners and costs; financing gaps visible; high-risk services protected.	Rushing into capital procurement before validation; reform fatigue; political disagreement.
Phase 2: Institutional foundations and targeted scale-up (2027)	Launch priority district/state compacts; strengthen contract management, workforce registries, facility registry, regulatory functions and interoperable data standards; pilot strategic purchasing and financial-protection mechanisms.	Model contracts; HRH establishment; regulatory implementation plans; enterprise architecture; partner-alignment scorecard.	Improved budget execution, staffing, reporting concordance, regulatory registration and essential-service reliability in pilot areas.	Pilots becoming isolated partner projects; inadequate recurrent financing; excessive indicator burden.
Phase 3: Expansion with verification (2028)	Expand proven service and purchasing models; integrate donor-supported workforce and systems; prioritise hospital/referral investments that pass readiness criteria; strengthen laboratory and emergency networks.	Scale-up decisions based on independent verification; recurrent-cost approvals; revised resource allocation.	Effective coverage and quality improve without rising financial hardship; reduced parallel systems; stronger referral completion.	Construction-led expansion; inequitable geographic allocation; funding volatility.
Phase 4: Consolidation and transition (2029)	Institutionalise annual public review; evaluate NTP outcomes and implementation mechanisms; transition to successor plan; protect financing and workforce gains; close or redesign ineffective initiatives.	NTP final implementation report; independent evaluation; successor health strategy and medium-term expenditure framework.	Core functions financed and governed through national/subnational institutions; transparent lessons and remaining gaps.	Overstating success against activity counts; loss of gains during strategy transition.

**Foundation 1: A federal-state governance compact.** A formal compact should clarify authority, shared functions, financing responsibilities, data obligations, performance review and dispute resolution. It should be endorsed politically and translated into operational terms for national, state, regional and district levels.

**Foundation 2: A costed and sequenced delivery plan.** Every target should be linked to an intervention package, geographic prioritisation, implementation dependency, cost, financing source, procurement route, owner and annual milestone. The plan should contain minimum, expected and accelerated financing scenarios.

**Foundation 3: Financing alignment and strategic purchasing.** Domestic and external financing should be mapped to one plan. Transitional pooled or pooled-equivalent mechanisms should use common results and audit frameworks. Provider contracts should purchase EPHS outputs and quality rather than reimburse fragmented project inputs.

**Foundation 4: Workforce, regulation and supply systems.** Workforce establishment, payroll, deployment, accreditation, supervision, medicines regulation, procurement and supply-chain functions should be developed as mutually dependent systems. Expansion should be paced by recurrent financing and institutional capacity.

**Foundation 5: Data, monitoring and accountability.** A national information architecture and limited core indicator set should support quarterly course correction and annual public accountability. Financial, service, quality and equity data should be reviewed together, with private and partner-supported services included.

**Foundation 6: Resilience and adaptive learning.** Routine programmes should incorporate risk assessment, contingency financing, emergency procurement, decentralised capacity, climate-resilient infrastructure, staff protection, community engagement and documented learning. Adaptive changes should be transparent and evidence-based.

## 7 Actionable recommendations and actor-specific responsibilities

Implementation failure often occurs because collective language such as ‘government and partners should’ obscures responsibility. Table 6 assigns primary accountabilities while recognising that many activities require joint action.

**Table 6:** Actor-specific priority actions for NTP health implementation.

Actor	Primary responsibilities	2026-2027 priority deliverables	Accountability mechanism
Federal Ministry of Health	National standards, consolidated planning, EPHS specification, regulation policy, national surveillance, partner coordination and sector review.	Validated implementation annex; HSSP IV; national indicator and facility registry; model service-purchasing arrangements; annual sector report.	Parliamentary/ministerial reporting, joint annual review, public dashboard and independent audit.
Federal Member State ministries and Banadir	Contextualised planning, facility and provider oversight, workforce deployment, supervision, district performance and community engagement.	State/region costed plans; facility readiness baselines; deployment and supervision plans; district review structures.	Quarterly performance review, community feedback, public state reports and joint verification.
Ministry of Finance and planning institutions	Fiscal pathway, budget credibility, expenditure tracking, intergovernmental financing and integration with national planning.	Medium-term health expenditure framework; protected priority lines; release and execution dashboard; financing-gap analysis.	Budget execution reports, audit and publication of health allocations/transfers.
Development partners and donors	Align financing to one plan, reduce parallel systems, provide predictable funding and invest in public institutional capacity.	Comprehensive financing map; common reporting and audit framework; multi-year commitments; transition plans for partner-funded staff/systems.	Annual partner-alignment scorecard and mutual accountability review.
Public, private and NGO providers	Deliver defined services and quality standards; report data; ensure referral, patient safety and financial transparency.	Registration and licensing; standard contracts/service agreements; quality-improvement plans; routine reporting.	Contract verification, licensing inspections, complaints data and performance disclosure.
Professional councils and medicines regulator	Registration, accreditation, product and facility regulation, inspection, ethics and enforcement.	Digital registries; risk-based inspection schedules; published standards and decisions; complaints/enforcement procedures.	Independent governance, public annual report, conflict-of-interest disclosure and appeal process.
Communities and civil society	Participate in priority setting, monitor access and quality, represent vulnerable groups and support trust and demand.	Facility committees, community scorecards, inclusive feedback and public hearings.	Documented response to complaints and inclusion in annual sector review.
Universities and research institutions	Implementation research, independent evaluation, workforce education, evidence synthesis and capacity development.	Priority implementation research agenda; embedded evaluation partnerships; accredited competency-based curricula.	Open protocols/results, research-use tracking and disclosure of funding/conflicts.

## 8 Discussion

### 8.1 Principal interpretation

The NTP creates an important political opportunity, but Somalia's main implementation deficit is institutional rather than conceptual. The sector already has multiple strategies, packages, roadmaps, investment cases and partnership commitments. The next advance will come from reconciling them into a limited, costed and governed implementation programme. The review identifies six foundations that determine whether activities become durable system functions: governance compact, operational sequencing, financing and purchasing, workforce and regulation, data and accountability, and resilience with adaptive learning.

The NTP should therefore not be assessed primarily by whether all announced infrastructure and numerical targets are completed. A more meaningful test is whether essential services become more reliable and equitable; whether public institutions can plan, finance, purchase, regulate and learn; whether households face less financial hardship; whether partner-supported staff and systems are progressively institutionalised; and whether services continue during shocks. This shifts attention from project completion to system capability.

### 8.2 Managing ambition and feasibility

Ambition is necessary in a setting with severe unmet need, but excessive simultaneous priorities can dilute implementation. The NTP combines major expansion of primary care, maternal and child health, immunisation, nutrition, hospitals, specialist services, workforce, insurance, domestic production, regulation, digital systems and emergency infrastructure. Somalia is unlikely to implement all components at the same pace. Sequencing should be guided by health impact, equity, feasibility, recurrent-cost sustainability, institutional dependencies and resilience value.

The first-order priorities are those that protect life and enable the rest of the plan: reliable EPHS delivery, maternal and newborn referral capacity, immunisation and nutrition continuity, essential medicines, funded frontline workers, surveillance and emergency response, facility functionality, and basic governance and information systems. Specialist and capital-intensive investments should proceed where population need, referral demand, operating finance and workforce are demonstrated. Insurance expansion should be approached as health-financing reform rather than enrolment promotion; without effective pooling, purchasing and subsidies, enrolment figures can overstate financial protection.

### 8.3 Political economy and incentives

Implementation is shaped by incentives. Federal and state institutions may compete over authority and resources; donors may favour visible, attributable projects; providers may resist reporting or price regulation; training institutions may expand enrolment beyond workforce demand; and communities may distrust services that are intermittently available or charge unpredictable fees. A technically strong plan that ignores these incentives will underperform.

The governance compact, financing map, provider contracts and public performance review are therefore political-economy instruments as much as management tools. They make resource flows and responsibilities visible, create negotiated expectations and provide mechanisms to resolve disputes. Independent verification and civil-society participation are important where institutions both implement and judge their own performance. Power analysis should be incorporated into major reforms, particularly regulation, purchasing, workforce payroll integration and geographic allocation (28, 58).

### 8.4 Implications beyond Somalia

This review contributes to wider health-system reform debates in fragile and conflict-affected settings. National transformation plans often combine developmental ambition with humanitarian dependence. The proposed readiness framework suggests that reform should be treated as a transition in system functions: from projects to institutions, fragmented financing to aligned purchasing, unregistered providers to governed networks, reporting to learning, and emergency substitution to resilient routine systems. These transitions are gradual and may use hybrid arrangements for extended periods.

The Somalia case also shows why country ownership should not be measured only by the proportion of funds passing through government accounts. Ownership includes authority to set priorities, access to complete financing information, control of standards, ability to purchase and regulate services, stewardship of national data, and capacity to sustain core functions. Transitional fiduciary safeguards can coexist with country leadership when they are explicitly designed to build rather than bypass institutions.

### 8.5 Strengths and limitations

The review's strengths are its integration of the full NTP health architecture, recent Somalia-specific evidence on maternal-service access, facility readiness, IPC, health-information supervision, AMR and climate-sensitive health risks, national policy documents, implementation-science concepts and comparative fragile-state evidence. It also identifies internal technical issues in the policy and converts broad recommendations into phased and actor-specific actions.

Several limitations should be recognised. This is a structured narrative review, not a systematic review, and some relevant unpublished or non-English material may have been missed. Publicly available implementation evidence remains limited because the NTP is in its early implementation period. National indicators vary across sources and years, and data from inaccessible or insecure areas may be incomplete. Comparative examples are context-dependent and cannot establish that the same reform will produce equivalent outcomes in Somalia. Finally, the framework is a policy-analysis product and should be validated through consultation with federal, state, Banadir, provider, partner and community stakeholders before formal adoption.

## 9 Conclusion

Somalia's National Transformation Plan provides a potentially important political platform for health-system reform, but its success will depend on implementation institutions and sustained political agreement. The evidence supports immediate technical validation of targets, protection of essential services and clearer governance, financing, workforce, regulatory, information and resilience arrangements. The six-foundation framework is a prospective proposal and should be tested through stakeholder consultation and implementation research before formal adoption.

A phased approach is therefore recommended: technical validation and a successor sector strategy in 2026; establishment and testing of governance, financing, workforce, regulatory and information foundations in 2027; evidence-dependent expansion in 2028; and consolidation and independent evaluation in 2029. Success should be judged by reliable essential care, improved financial protection, accountable institutions and continuity during shocks.
