# Supplementary material for: Implementing Somalia's National Transformation Plan 2025–2029 for health-system reform: a policy and practice review of governance, financing, and institutional readiness
Source: Front Health Serv. 2026 Sep 30;6:1934347. doi: 10.3389/frhs.2026.1934347 (PMC13662005; doi:10.3389/frhs.2026.1934347)
Supplement: Supplementary file 1 [file Supplementaryfile1.docx]

Supplementary Material

Implementing Somalia's National Transformation Plan 2025-2029 for Health-System Reform: A Policy and Practice Review of Governance, Financing, and Institutional Readiness

# Supplementary Table S1. Search concepts and source strategy

The search was designed for a structured critical narrative policy review rather than a systematic intervention-effect review. Searches were conducted from June to July 2026 using Google Scholar as the multidisciplinary scholarly index, PubMed, PubMed Central, targeted publisher searches and institutional repositories. Peer-reviewed evidence was combined with authoritative national policy documents and institutional reports because the review question concerns policy content, implementation conditions and institutional readiness. Comparative searches covered Afghanistan, Ethiopia, Liberia, Rwanda and Sierra Leone. South Sudan was not retained because no eligible South Sudan source contributed to the final synthesis.

| **Source group** | **Core concepts** | **Illustrative search string** | **Eligibility emphasis** | **Purpose** |
| --- | --- | --- | --- | --- |
| National policy architecture | Somalia; NTP; NDP-9; HSSP III; EPHS; UHC; implementation | Somalia AND ("National Transformation Plan" OR NDP-9 OR HSSP OR EPHS OR UHC) AND (implementation OR governance OR financing) | Current or foundational national policies; official strategies; implementation and investment documents. | Map commitments, institutional responsibilities, indicators and policy coherence. |
| Governance, federalism and regulation | Somalia; federalism; decentralisation; stewardship; regulation; private sector | Somalia AND (federalism OR decentralization OR stewardship OR regulation OR "private providers") AND health | Somalia-specific empirical studies, policy analyses and authoritative reports. | Assess authority, coordination, regulation and mixed-provider governance. |
| Financing and purchasing | Somalia; domestic financing; aid; out-of-pocket; insurance; pooling; purchasing | Somalia AND ("health financing" OR aid OR "out-of-pocket" OR insurance OR pooling OR purchasing) | National expenditure evidence; household financial-protection studies; FCAS financing evidence. | Assess fiscal feasibility, financial protection, alignment and purchasing capacity. |
| Workforce, information and delivery capacity | Somalia; workforce; community health; DHIS2; HMIS; digital health; service quality | Somalia AND ("health workforce" OR "community health worker" OR DHIS2 OR HMIS OR "digital health" OR quality) | Empirical and implementation evidence relevant to delivery capacity. | Assess workforce, information, quality-management and service-delivery readiness. |
| Fragile-state comparison | fragile/conflict affected; health reform; contracting; pooled financing; resilience | ("fragile and conflict-affected" OR post-conflict) AND (health-system reform OR contracting OR financing OR resilience) | Systematic/scoping reviews and well-documented country experiences with clear mechanisms. | Identify transferable mechanisms and contextual cautions. |

# Supplementary Table S2. Evidence charting and critical interpretation

| **Citation/document** | **Source type** | **Domain** | **Principal evidence or commitment** | **Implication for NTP implementation** | **Important limitation** |
| --- | --- | --- | --- | --- | --- |
| Federal Republic of Somalia, NTP 2025-2029 [1] | National development policy | Overall reform architecture | Sets quantified ambitions for UHC, financing, workforce, infrastructure, regulation, digitalisation and emergency preparedness. | Provides political mandate, but requires technical validation, costing, sequencing and assignment of accountable institutions. | Some targets and baselines require reconciliation; high-level commitments do not establish implementation feasibility. |
| Federal MoH, HSSP III 2022-2026 [2] | National sector strategy | Sector governance and delivery | Defines sector priorities around EPHS, governance, workforce, financing and information systems. | The 2026 transition should align HSSP successor arrangements and annual plans with the NTP rather than create parallel planning tracks. | Predates the NTP and reaches the end of its implementation period during the NTP cycle. |
| Federal MoH, Health Sector Investment Case 2022-2027 [4] | Costing and investment document | Financing and priority interventions | Identifies priority EPHS interventions and resource requirements. | Offers a foundation for NTP costing, resource mapping and financing-gap analysis. | Cost assumptions, prices, donor commitments and service-delivery conditions require updating. |
| Somali National Bureau of Statistics, SHDS 2020 [6] | National household survey | Need, equity and baseline indicators | Documents major maternal, child, nutrition, access and regional inequalities. | Supports geographic and population prioritisation and provides a baseline for equity-sensitive implementation. | Data precede the NTP and may incompletely capture insecure, mobile and displaced populations. |
| Jama et al., 2023 [10] | WHO practice report | EPHS and stakeholder alignment | Shows that inclusive stakeholder engagement enabled agreement on a prioritised, progressively realised EPHS. | Use the EPHS as the common service, costing, contracting and monitoring unit across funders and providers. | Describes a policy-development process rather than measured implementation outcomes. |
| Said and Kicha, 2024 [11] | Somalia health-system policy analysis | Federalism and governance | Highlights overlapping responsibilities, fiscal constraints and federal-state implementation challenges. | A negotiated federal-state compact is needed to define functions, standards, reporting and escalation pathways. | Narrative analysis; does not empirically test governance reforms. |
| Dirie et al., 2025 [13] | Qualitative multi-stakeholder study | PHC, workforce and regulation | Stakeholders prioritised community-centred PHC, workforce development, regulatory strengthening and digital partnerships. | Provides contextual validation for sequencing reforms around frontline delivery capacity and institutional governance. | Thirty purposively selected participants; findings are not nationally representative. |
| Nikoloski et al., 2025 [14] | Somalia UHC policy analysis | UHC and financing | Identifies governance, financing and service-delivery constraints affecting UHC in a fragile context. | NTP monitoring should link service coverage to quality, equity and financial protection rather than report access alone. | Depends on available national and institutional data, which vary in completeness and recency. |
| Ahmed et al., 2020 [18] | Mixed-methods Somalia case study | Humanitarian-development interface | Documents donor priorities, insufficient and inflexible funding, workforce and commodity shortages, insecurity and access barriers. | Protect essential services and align humanitarian and development financing during the transition to national systems. | Evidence reflects the humanitarian response since 2000 and may not capture all recent institutional changes. |
| Mohamud et al., 2026 [17] | Household health-financing study | Financial protection | Reports high out-of-pocket spending, very limited insurance and substantial catastrophic expenditure. | Prioritise publicly financed protection for poor households and strategic purchasing before pursuing rapid contributory insurance expansion. | Authors should verify sampling, weighting and national representativeness when interpreting point estimates. |
| Hussein et al., 2026 [16] | Structured narrative review | Aid dependence and governance | Finds that external assistance expanded services but sustained parallel financing, supply and data arrangements. | Use pooled-equivalent alignment, system integration and gradual payroll transition to reduce dependency without disrupting services. | Synthesises heterogeneous peer-reviewed and grey evidence; causal attribution is limited. |
| Mothupi et al., 2025 [19] | Qualitative policy case study | Political prioritisation and accountability | Finds technical policy progress alongside weak domestic ownership, fragmented accountability and strong external influence. | Implementation needs political championship, inclusive decision-making and explicit mutual accountability. | Federal-level stakeholder study; subnational perspectives require further validation. |
| Bogale et al., 2024 [20] | Review of systematic reviews | FCAS health-system strengthening | Finds that effective strengthening is multicomponent, coordinated and adapted to the stage and context of fragility. | Avoid isolated infrastructure or training interventions; combine governance, workforce, financing, community and information reforms. | Underlying reviews and country settings are heterogeneous. |
| Truppa et al., 2024 [21] | Systematic scoping review | Health-system resilience | Governance-centred approaches predominate; transformative capacity and community roles are underdocumented. | Embed adaptive learning, continuity planning, community participation and transformation indicators in NTP governance. | Broad FCAS evidence is not specific to Somalia and does not establish comparative effectiveness. |

# Supplementary Table S3. Technical validation requirements for selected NTP targets

| **Target area** | **NTP commitment or reported baseline** | **Technical validation required** | **Suggested implementation metric** | **Interpretive caution** |
| --- | --- | --- | --- | --- |
| UHC service coverage | Increase the UHC service-coverage index from 33.5 (2021 estimate) to 40 by 2029 [1,8]. | Confirm baseline year, tracer composition, data source, subnational disaggregation and relationship to effective coverage. | Annual national and FMS tracer coverage, quality-adjusted coverage and service-use equity gaps. | A higher service-coverage index does not by itself demonstrate quality or financial protection. |
| Government health spending | Increase government health spending to 10% of the national budget [1]. | Define annual nominal and percentage pathways, releases, execution rates, protected recurrent lines and federal/FMS contributions. | Budget share, per-capita public spending, release rate, execution rate and share reaching frontline services. | The target is below the Abuja 15% benchmark and neither benchmark proves resource adequacy or efficient use. |
| Hospital infrastructure | Rehabilitate 18 regional hospitals and establish state referral capacity; the NTP reports seven referral hospitals in an earlier summary and eight in the detailed health chapter [1, Chapter 15, pp. 425-435]. | Reconcile the number, locations, service specifications, capital costs, staffing, maintenance and referral-network assumptions. | Functional readiness score, core specialist services, referral completion, operating cost and quality indicators. | Construction without workforce, supplies, utilities, maintenance and referral systems may not improve access or outcomes. |
| Health workforce | Increase staffing and fill priority posts from a reported planning baseline of 7.91 health workers per 10,000 population [1]. | Confirm cadre definitions, funded establishment, payroll status, competency requirements, distribution and retention assumptions. | Filled funded posts, payroll integration, rural retention, competency verification and workforce density by cadre/location. | Headcounts can conceal absenteeism, skill gaps, urban concentration and unstable partner-funded positions. |
| Health insurance and financial protection | Expand health-insurance penetration to 35% by 2029; the source plan does not define the denominator or financing design [1, Chapter 15, pp. 425-435]. | Define scheme type, population entitlement, benefit package, subsidies, revenue source, pooling, purchasing and grievance mechanisms. | Population legally entitled and actively covered; claims/use; OOP share; catastrophic and impoverishing expenditure. | Rapid voluntary contributory insurance may exclude poor and informal households and fragment risk pools. |
| DTP3 immunisation | Increase DTP3 coverage to 80% by 2029; the 2024 WHO/UNICEF estimate was 42% [1, Chapter 15, pp. 425-435; 25]. | Verify baseline source/year, target population denominator, administrative/WUENIC differences, zero-dose burden and dropout. | DTP1, DTP3, DTP1-DTP3 dropout, zero-dose prevalence, district equity and stock-out days. | Administrative coverage may be distorted by uncertain denominators, displacement and cross-district service use. |
| Maternal and child mortality | Reduce maternal mortality from 692 to <400 per 100,000 live births; neonatal mortality from 38 to <20; infant mortality from 61 to <37; and under-five mortality from 122 to <60 per 1,000 live births by 2029 [1,6,7]. | Specify measurement sources, uncertainty intervals, reporting frequency and intermediate service/quality indicators. | Mortality estimates plus skilled birth attendance, EmONC readiness, referral time, neonatal care quality and avoidable-death review. | Mortality estimates change slowly and may not support annual attribution to NTP activities. |
| Regulation and digital systems | Operationalise professional, medicines, facility and digital-health governance. | Confirm legal mandates, institutional financing, registries, licensing/inspection standards, interoperability and data-protection rules. | Share licensed/inspected; enforcement actions resolved; essential systems interoperable; data completeness, timeliness and use. | Creating institutions or platforms without recurrent financing, enforcement authority and user capacity may produce nominal compliance only. |

# Supplementary Table S4. Minimum evidence package for annual NTP health-sector review

| **Domain** | **Minimum evidence** | **Required disaggregation** | **Decision enabled** |
| --- | --- | --- | --- |
| Financing | Approved budgets, releases, expenditure, partner commitments, off-budget flows, provider payments and arrears. | Federal/FMS/Banadir; programme; source; recurrent/capital; facility where feasible. | Reprioritise financing, address bottlenecks and protect essential recurrent inputs. |
| Service access and quality | EPHS service volume and coverage, readiness, stock-outs, referral completion, waiting times and selected quality/outcome indicators. | District/facility; sex; age; displacement; residence; disability; wealth proxy where feasible. | Identify service gaps, redeploy resources and trigger quality-improvement support. |
| Workforce | Funded and filled posts, payroll status, attendance, competencies, training, attrition and distribution. | Cadre; gender; employer/funder; facility; rural/urban; FMS/Banadir. | Approve recruitment, payroll transition, redistribution, supervision and retention actions. |
| Governance and regulation | Compact compliance, contracts, inspections, licensing, audit findings, grievances and corrective-action closure. | Institution; provider type; geography; risk level. | Escalate non-compliance, adjust contracts and strengthen regulatory enforcement. |
| Information and learning | Completeness, timeliness, concordance, interoperability, data-use decisions and implementation-research findings. | System; indicator; facility; geography; reporting entity. | Correct data problems, revise indicators and adapt implementation design. |
| Equity and resilience | Coverage and quality gaps, disruption days, emergency activation, climate/security risks, continuity and recovery. | Vulnerable population; geography; shock type; service. | Protect marginalised groups, pre-position resources and revise continuity plans. |

## Supplementary Table S5. Search screening and source-selection record

| **Review element** | **Procedure used** |
| --- | --- |
| Search dates | From June to July 2026. |
| Scholarly sources | Google Scholar, PubMed, PubMed Central and targeted publisher searches. |
| Grey-literature sources | Federal Government of Somalia, Federal Ministry of Health and Human Services, Ministry of Planning, Somali National Bureau of Statistics, WHO, UNICEF, World Bank, Global Financing Facility and related institutional repositories. |
| Comparative scope | Afghanistan, Ethiopia, Liberia, Rwanda and Sierra Leone. South Sudan was removed because no eligible source from that setting was retained. |
| Eligibility | English-language sources published primarily from 2015 to 2026 that addressed Somalia's health policies, implementation conditions or relevant fragile-setting experience; earlier foundational sources were retained when conceptually necessary. |
| Screening | Titles, abstracts or executive summaries were screened first, followed by full-text assessment. Sources were excluded when provenance could not be verified, when they duplicated a more authoritative source or when they did not directly inform implementation. |
| Reviewers | One author conducted initial screening and evidence charting. Potentially eligible sources, domain assignments and interpretive differences were checked by the author team and resolved by consensus. |
| Final corpus | The final purposive corpus comprised 60 cited sources. |
| Yield accounting | Database-level yield counts were not retained because the search was purposive and iterative. Accordingly, no PRISMA flow diagram is presented; this is acknowledged as a limitation of the structured narrative review. |

## Supplementary Table S6. Derivation of the proposed six readiness foundations

| **Proposed foundation** | **Evidence-charting domains** | **Analytical contribution** | **Status and validation need** |
| --- | --- | --- | --- |
| 1. Federal-state governance compact | Policy coherence; federal and institutional governance; private-sector and partner engagement | The health-policy triangle connected policy content with actors, context and process; CFIR highlighted outer-setting relationships and implementation process. | Analytical proposal requiring consultation with federal authorities, FMS, Banadir, providers, partners, civil society and communities. |
| 2. Costed and sequenced delivery plan | Policy coherence; service delivery and infrastructure; financing and purchasing | CFIR's innovation domain focused attention on the operational package, its design quality, dependencies and implementation sequence. | Prospective planning foundation; not an empirically validated causal requirement. |
| 3. Financing alignment and strategic purchasing | Financing and purchasing; private-sector and partner engagement; governance | The policy triangle clarified resource and actor incentives, while comparative evidence informed cautious options for pooling, alignment and contracting. | Requires fiscal, fiduciary, equity and provider-market testing before scale-up. |
| 4. Workforce, regulation and supply systems | Workforce and regulation; service delivery and infrastructure; governance | CFIR inner-setting and individual domains linked staffing, competencies, organisational capacity, regulation and supply reliability. | Requires verified workforce, regulatory and supply-chain baselines and staged implementation testing. |
| 5. Data, monitoring and accountability | Data, monitoring and accountability; policy coherence; partner engagement | CFIR implementation process and the policy triangle supported feedback, transparent responsibility, data use and public accountability. | Core indicators and accountability arrangements require stakeholder agreement and field validation. |
| 6. Resilience and adaptive learning | Emergency preparedness and resilience; service delivery; financing; data and learning | The resilience lens assessed absorptive, adaptive and transformative capacity under recurrent shocks and linked routine and emergency functions. | Prospective proposal requiring validation during routine implementation and real or simulated shocks. |
